# Biotransformation of Timosaponin BII into Seven Characteristic Metabolites by the Gut Microbiota

**DOI:** 10.3390/molecules26133861

**Published:** 2021-06-24

**Authors:** Guo-Ming Dong, Hang Yu, Li-Bin Pan, Shu-Rong Ma, Hui Xu, Zheng-Wei Zhang, Pei Han, Jie Fu, Xin-Yu Yang, Adili Keranmu, Hai-Tao Niu, Jian-Dong Jiang, Yan Wang

**Affiliations:** 1 Beijing Hwellso Pharmaceutical Co., Ltd., Beijing 100044, China; qcdgm@126.com; 2 State Key Laboratory of Bioactive Substance and Function of Natural Medicines, Institute of Materia Medica, Chinese Academy of Medical Sciences/Peking Union Medical College, Beijing 100050, China; yuhang@imm.ac.cn (H.Y.); panlibin@imm.ac.cn (L.-B.P.); mashurong@imm.ac.cn (S.-R.M.); xuhui@imm.ac.cn (H.X.); zhangzhengwei@imm.ac.cn (Z.-W.Z.); hanpei@imm.ac.cn (P.H.); fujie@imm.ac.cn (J.F.); xinyuy20@mails.jlu.edu.cn (X.-Y.Y.); adili117@163.com (A.K.); 3 School of Medicine and Institute of Laboratory Animal Sciences, Jinan University, Guangzhou Key Laboratory of Germ-free Animals and Microbiota Application, Guangzhou 510632, China; htniu@jnu.edu.cn

**Keywords:** timosaponin BII, gut microbiota, metabolites, LC-MS/MS, LC/MS-Q-TOF

## Abstract

Timosaponin BII is one of the most abundant Anemarrhena saponins and is in a phase II clinical trial for the treatment of dementia. However, the pharmacological activity of timosaponin BII does not match its low bioavailability. In this study, we aimed to determine the effects of gut microbiota on timosaponin BII metabolism. We found that intestinal flora had a strong metabolic effect on timosaponin BII by HPLC-MS/MS. At the same time, seven potential metabolites (M1–M7) produced by rat intestinal flora were identified using HPLC/MS-Q-TOF. Among them, three structures identified are reported in gut microbiota for the first time. A comparison of rat liver homogenate and a rat liver microsome incubation system revealed that the metabolic behavior of timosaponin BII was unique to the gut microbiota system. Finally, a quantitative method for the three representative metabolites was established by HPLC-MS/MS, and the temporal relationship among the metabolites was initially clarified. In summary, it is suggested that the metabolic characteristics of gut microbiota may be an important indicator of the pharmacological activity of timosaponin BII, which can be applied to guide its application and clinical use in the future.

## 1. Introduction

Anemarrhena saponins are steroidal saponins derived from the plant *Anemarrhena asphodeloides* Bge of the family Liliaceae. Studies have shown that Anemarrhena saponins have a variety of pharmacological activities, including effects on the symptoms of dementia, improvements in learning and memory, remission of depression, vascular protection, anticoagulant and antithrombotic activities, regulation of glucose and lipid metabolism, and antitumor activities [1,2,3,4,5,6,7]. At present, more than 40 Anemarrhena saponins have been reported and widely exist in *Anemarrhena asphodeloides* Bge [8]. As the saponin with the highest content in *Anemarrhena asphodeloides* Bge., timosaponin BII has been a focus of research in recent years; its structure is (25S)-26-O-β-D-glucopyranosyl-22-hydroxy-5β-furostane-3β,26-diol-3-O-β-D-glucopyranosyl-(1→2)-β-D-galactopyranoside [9]. Timosaponin BII has been reported to have pharmacological activities such as effects on the symptoms of dementia, inhibiting platelet aggregation, anti-inflammatory activity, and improving learning and memory functions [4,10,11,12]. Currently, timosaponin BII is in a clinical trial for the treatment of dementia. Nevertheless, the absolute bioavailability of timosaponin BII is relatively low, only approximately 1.1% (rats) [13], which suggests that timosaponin BII may undergo extensive metabolism after oral administration. An in vitro incubation experiment using simulated gastric fluid and simulated intestinal fluid showed that Anemarrhena saponins may undergo a hydrolysis reaction to generate hydrolyzed metabolites [14]. It has been shown that not only the parent drug of timosaponin BII itself, but also its metabolites may have pharmacological activity.

The gut microbiome includes trillions of cells and more than 1000 species of bacteria, fungi and phages that reside in the human intestine, which houses the core part of the intestinal microecosystem [15,16]. Intestinal flora interacts with host cells via the communication of material, energy and even genes and carries out important and diverse physiological functions for its host. As the second genomes of human beings, the gut microbiota affects human health and disease development and has been considered as the “forgotten invisible organ of the human body” [17,18,19]. The gut microbiota is involved in the occurrence and development of many diseases, and the regulation of intestinal flora has become an important target for the treatment of diseases [20,21]. The intestinal flora is rich in a large number of enzymes related to material metabolism [22]. Therefore, gut microbiota plays important roles in the biotransformation and bioavailability of natural oral medicines [23]. In contrast to liver metabolism, the type of biotransformation caused by intestinal flora is unique, which mainly includes hydrolysis and reduction reactions [22,24,25,26]. It has been reported that some fungi, such as *Colletotrichum gloeosporioides*, *Acremonium alternatum*, and *Aspergillus niger*, can be used for the biotransformation of Anemarrhena saponins [27], but this biotransformation requires supplementation with exogenous glucose. In addition, after timosaponin AIII was cocultured with *Saccharomyces cerevisiae* for 24 h, five metabolites were obtained [28]. At present, there are few reports directly studying the interaction between intestinal flora and Anemarrhena saponins, and further research is required.

In this study, the metabolism of timosaponin BII by intestinal flora was explored by high-performance liquid chromatography coupled with tandem mass spectrometry (HPLC-MS/MS) and high-performance liquid chromatography coupled with quadrupole time-of-flight mass spectrometry (HPLC/MS-Q-TOF), and the possible mass spectrometry cleavage pathways of metabolites were summarized. In addition, timosaponin BII was incubated with the liver microsome system and the liver homogenate system to compare the metabolic capacity of the gut microbiota. Seven possible metabolites of timosaponin BII produced by intestinal flora were identified. Among them, three structures were reported in the gut microbiota for the first time. Moreover, the possible metabolic pathways for the three kinds of metabolites were revealed for the first time. This research furthers the elucidation of the metabolism of Anemarrhena saponins by gut microbiota and thus may provide new insight for basic research on the efficacy of saponins and future applications.

## 2. Results

### 2.1. Timosaponin BII Was Significantly Metabolized by the Gut Microbiota

**Figure 1:**
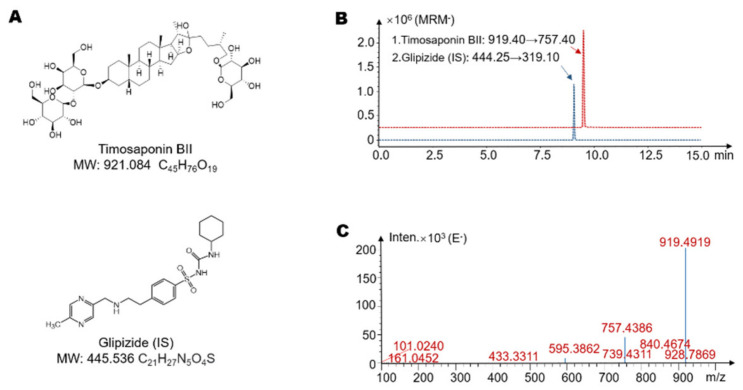
The structure and mass spectra of timosaponin BII. (**A**) The structural formula of timosaponin BII and the Glipizide (internal standard (IS)). (**B**) Extracted ion chromatogram (EIC) spectra of timosaponin BII and the internal standard (Glipizide). (**C**) The high-resolution mass spectra of timosaponin BII acquired by LC/MS-Q-TOF.

In this study, we aimed to clarify whether timosaponin BII can interact with gut microbiota to produce a unique metabolic profile. By comparing the metabolic pathways of intestinal flora with those of the liver, we wanted to indicate the unique role of intestinal flora in drug metabolism. Figure 1A shows the molecular structure of timosaponin BII and the molecular structure of the internal standard, glipizide. A method for quantifying timosaponin BII was established using HPLC-MS/MS to explore whether timosaponin BII can be metabolized by gut microbiota. The retention time of timosaponin BII in this method was 9.5 min, and the retention time of the internal standard was 9.1 min. A chromatogram generated with the established method is shown in Figure 1B. In addition, we used HPLC/MS-Q-TOF to identify timosaponin BII, and its high-resolution mass spectrum is shown in Figure 1C. The molecular ion of timosaponin BII existed in the form of [M − H]^−^, with *m*/*z* of 919.4919.

**Figure 2:**
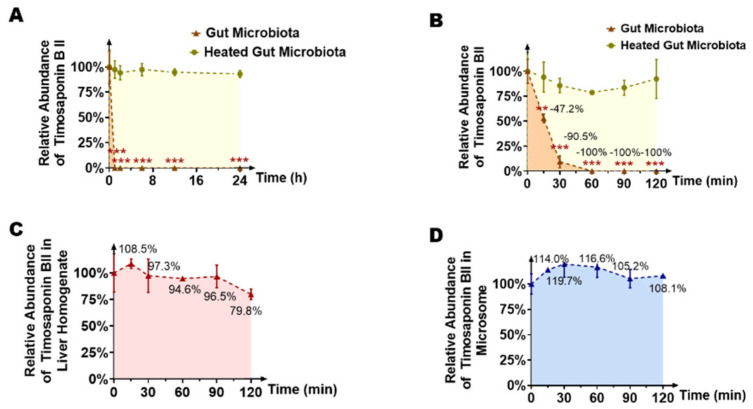
Timosaponin BII could be metabolized in the gut microbiota. Intestinal bacteria mainly participated in the metabolism of timosaponin BII by comparison between untreated and heated intestinal contents (**A**,**B**). (**A**) The level of timosaponin BII decreased during incubation with rat intestinal bacteria after 0 h, 1 h, 2 h, 6 h, 12 h, and 24 h. (**B**) The metabolic characteristic of timosaponin BII by gut microbiota in more precise spans of time. The liver may not be the metabolic site of timosaponin BII (**C**,**D**). (**C**) The metabolic curve of timosaponin BII by rat liver homogenate. (**D**) The metabolic curve of timosaponin BII by rat microsome. Data are presented as mean ± SD, and two-tailed Student’s *t* test were used for analysis (** *p* < 0.01, *** *p* < 0.001).

Next, a mixture of the colon contents of seven SD rats was incubated with timosaponin BII (final concentration of 10 μg/mL) for 24 h to explore the metabolic effects of gut microbiota. An incubation system of the colon contents inactivated by heating twice was used as a negative control to eliminate the interference of environmental factors such as the culture medium. Samples were collected at 0 h, 1 h, 2 h, 6 h, 12 h and 24 h after incubation. The content of timosaponin BII in the system was detected by HPLC-MS/MS. As shown in Figure 2A, timosaponin BII disappeared rapidly in this system. Compared with the inactivated negative control, the concentration of the original drug was completely consumed by 1 h, while the concentration of the original drug remained in the inactivated control group at 24 h, which indicated the high extent of metabolism of timosaponin BII by intestinal flora. To further understand the effects of the metabolic characteristics of intestinal flora on timosaponin BII, the time span between sampling points was decreased. In the same incubation system, the concentration of timosaponin BII in the samples was detected at 0 h, 0.25 h, 0.5 h, 1 h, 1.5 h and 2 h after incubation. Figure 2B clearly shows that the timosaponin BII concentration showed a rapid downward trend over time: 47.2, 90.5 and 100% of the original drug had been metabolized by intestinal bacteria by 15 min, 30 min, and 1 h, respectively.

To explore whether the liver has a similar metabolic effect on timosaponin BII, we incubated timosaponin BII in rat liver homogenate or rat microsome system respectively, and the content of the original drug in the sample was detected at 0 h, 0.25 h, 0.5 h, 1 h, 1.5 h and 2 h (Figure 2C,D). Over 2 h of incubation, we found that the content of timosaponin BII in the liver homogenate showed a slightly downward trend: 108.5%, 97.3%, 94.6%, 96.5%, 79.8% of the origin concentration at 0.25 h, 0.5 h, 1 h, 1.5 h and 2 h. And microsome system did not change significantly: 114.0%, 119.7%, 116.6%, 105.2%, 108.1% of the origin concentration at 0.25 h, 0.5 h, 1 h, 1.5 h and 2 h, which indicated that liver may not be the main site of timosaponin BII metabolism.

### 2.2. Timosaponin BII Was Transformed to Seven Metabolites by the Gut Microbiota

**Figure 3:**
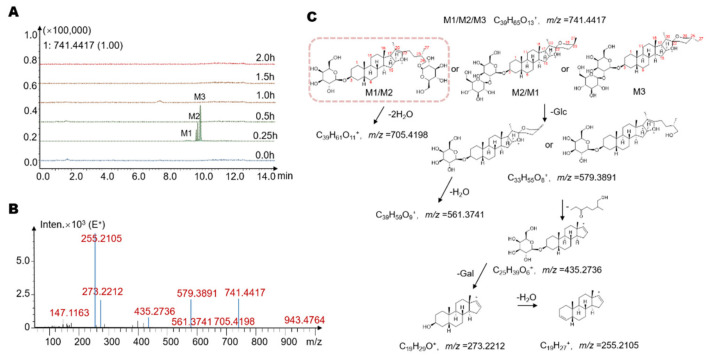
(**A**) The extracted ion chromatograms (EICs) showed that the levels of the possible metabolites M1–M3 increased with increasing time. (**B**) The MS/MS data of the timosaponin BII metabolites M1–M3. (**C**) Possible structures and mass spectrometric cleavage pathway of metabolites M1–M3. Among them, the structure framed by a rectangle was reported in the gut microbiota for the first time.

**Figure 4:**
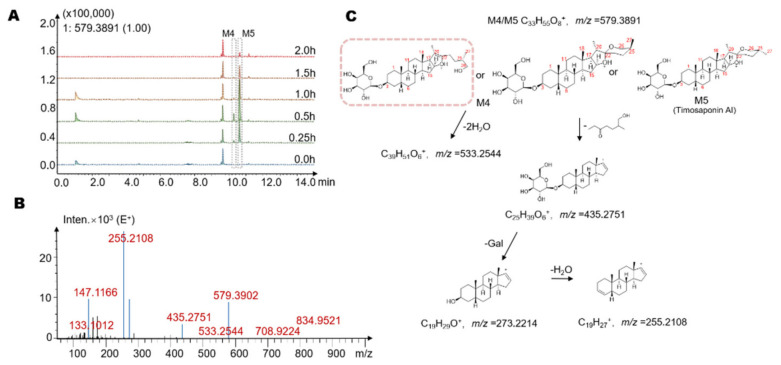
(**A**) The extracted ion chromatograms (EICs) showed that the levels of the possible metabolites M4 and M5 increased with increasing time. (**B**) The MS/MS data of the timosaponin BII metabolites M4 and M5. (**C**) Possible structures and mass spectrometric cleavage pathway of metabolites M4 and M5. Among them, the structure framed by a rectangle was reported in the gut microbiota for the first time.

**Figure 5:**
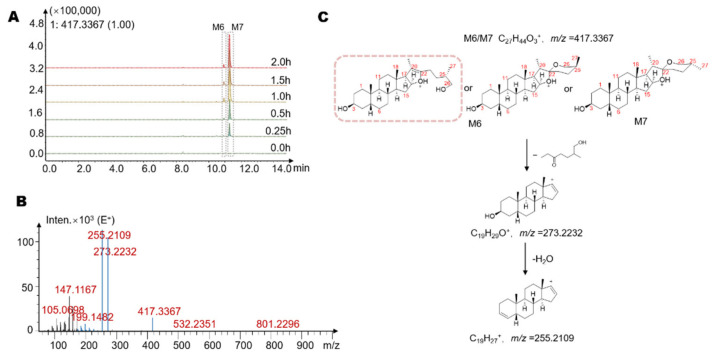
(**A**) The extracted ion chromatograms (EICs) showed that the levels of the possible metabolites M6 and M7 increased with increasing time. (**B**) The MS/MS data of the timosaponin BII metabolites M6 and M7. (**C**) Possible structures and mass spectrometric cleavage pathway of metabolites M6 and M7. Among them, the structure framed by a rectangle was reported in the gut microbiota for the first time.

To explore the metabolites of timosaponin BII produced by gut microbiota, we used HPLC/MS-Q-TOF to identify the suspected metabolites in the metabolic system. In this experiment, a total of seven potential metabolites were detected with retention times of 9.9 min, 10.1 min, 10.2 min, 10.3 min, 10.6 min, 10.9 min and 11.3 min (M1–M7, Figure 3, Figure 4 and Figure 5A, Table 1). Several metabolites showed different time-quantity relationships in the incubation system (see Section 2.3 for details). It is worth noting that these metabolites were not detected in the inactivated negative control group, indicating that M1–M7 are not produced via environmental effects but via the metabolism of the intestinal flora.

### 2.3. Identification of Seven Gut Microbiota-Metabolites by HPLC/MS-Q-TOF

Next, the possible structures of several metabolites were studied. The mass spectra of M1–M3 and possible structures are shown in Figure 3B,C. M1–M3 shared the same relative molecular weight, with a molecular formula of C_39_H_64_O_13_. The quasi-molecular ions of the three metabolites were all [M + H]^+^, with *m*/*z* = 741.4417. The retention times were 9.9 min, 10.1 min, and 10.2 min, respectively. The high-resolution mass spectra obtained from QTOF analysis are summarized as follows. The ion with *m*/*z*= 705.4198 was obtained by loss of 2 H_2_O from the quasi-molecular ion (M+H^+^-2H_2_O, *m*/*z* = 705.4198). The subsequent ion with *m*/*z* of 579.3891 was the product ion obtained from the quasi-molecular ion by loss of one glucose moiety (M+H^+^-C_6_H_10_O_5_, *m*/*z* = 579.3891), and the latter continued to lose 1 H_2_O to obtain the ion of M+H^+^-C_6_H_10_O_5_-H_2_O, with *m*/*z* = 561.3741. At the same time, the ion with *m*/*z* = 579.3891 can also lose the E ring with its side chain to obtain an ion with *m*/*z* = 435.2736 (M+H^+^-C_6_H_10_O_5_-C_8_H_16_O_2_, *m*/*z* = 435.2736). Finally, the ions with *m*/*z* of 273.2212 and 255.2105 were the product ions after the quasi-molecular ion lost all its side chains and its sugar moiety (M+H^+^-2 C_6_H_10_O_5_-C_8_H_16_O_2_, 273.2212) and further continued to lose 1 H_2_O (M+H^+^-2 C_6_H_10_O_5_-C_8_H_16_O_2_-H_2_O, 255.2105). Based on the molecular structure, molecular weight and mass spectral cleavage pathways of timosaponin BII and M1–M3, we speculated that M1–M3 may be isomers of timosaponin BII with 1 glucose moiety removed at position C_3_ or C_26_. After comparison with the chromatogram of the timosaponin AIII, we suspected that M3 might be timosaponin AIII. Among them, the structure framed in Figure 3C by a rectangle is reported in the gut microbiota for the first time.

In addition, we found M4 and M5, which have a smaller *m*/*z* of 579.3891 ([M + H]^+^) and retention times of 10.3 min and 10.6 min, respectively. The molecular formula of M4 and M5 was C_33_H_54_O_8_. The mass spectra of M4 and M5 and possible structures are shown in Figure 4B,C. The high-resolution mass spectra obtained from QTOF analysis are summarized as follows. The ion with *m*/*z* 533.2544 was obtained by loss of 2 H_2_O from the quasi-molecular ion (M+H^+^-2H_2_O, *m*/*z* = 533.2544). Similarly, the E ring with its side chain can also be cleaved from the quasi-molecular ion to obtain a product ion with *m*/*z* = 435.2751 (M+H^+^-C_8_H_16_O_2_, *m*/*z* = 435.2736), and the latter can then lose 1 glucose or galactose moiety followed by 1 H_2_O to yield a product ion with *m*/*z* = 255.2108. Based on the molecular weight and the structure of timosaponin BII, we believed that M3 and M4 were the hydrolysates of the parent drug generated by removal of two 6-carbon sugar moieties, which may occur via the removal of all the lactose chains at the C_3_ position or the removal of two glucose moieties at C_26_ and C_3_, respectively. However, we found that the ion with *m*/*z* = 435.2751 (M+H^+^-C_8_H_16_O_2_) was preferentially generated in the mass spectra of M3 and M4, indicating that the hydroxyl group at C_26_ had been freed. The ion M+H^+^-C_6_H_10_O_5_, which was the product ion produced from the quasi-molecular ion via a neutral loss of one glucose, was not found, suggesting that both M3 and M4 were metabolites obtained by removing two glucose moieties at C_26_ and C_3_. Thus, M4 and M5 may be stereoisomers at the C_27_ methyl group or ring-opening isomers at spiroalkane (Figure 4C). After comparison with the chromatogram of the timosaponin AI, we suspected that M5 might be timosaponin AI. Among them, the structure framed in Figure 4C by a rectangle is reported in gut microbiota for the first time.

We also found potential metabolites with a molecular formula of C_27_H_44_O_3_ at 10.9 min and 11.3 min, named M6 and M7 (*m*/*z* = 417.3367). The high-resolution mass spectra obtained from QTOF analysis are summarized as follows (Figure 5B,C). The ion with *m*/*z* = 273.2232 was the product ion yielded from the removal of the E ring with its side chain from the quasi-molecular ion (M+H^+^-C_8_H_16_O_2_, *m*/*z* = 273.2232). The ion of 255.2109 was the ion of the steroid structure, which was the product ion (M+H^+^-C_8_H_16_O_2_-H_2_O) produced by the loss of 1 H_2_O from M+H^+^-C_8_H_16_O_2_. Based on the molecular weight and the ion with *m*/*z* = 255.2109, it was considered that M6 and M7 were isomers of the aglycon part obtained by removing all sugar chains from timosaponin BII and may be stereoisomers at the C_27_ methyl group or ring-opening isomers at spiroalkane (Figure 5C). After comparison with the chromatogram of the sarsasapogenin, we suspected that M7 might be sarsasapogenin. Among them, the structure framed in Figure 5C by a rectangle is reported in gut microbiota for the first time.

### 2.4. Temporal Characteristics of Main Timosaponin BII Metabolites (Timosaponin AIII, Timosaponin AI and Sarsasapogenin) by Gut Microbiota

To explore the temporal sequence of the production of potential metabolites of timosaponin BII by intestinal flora, three representative metabolites, namely, timosaponin AIII (C_39_H_64_O_13_), timosaponin AI (C_33_H_54_O_8_) and sarsasapogenin (C_27_H_44_O_3_) (the corresponding structures are labeled in Figure 3, Figure 4 and Figure 5C), were purchased. We used an LC-MS 8060 system to establish a quantitative analysis method for the detection of timosaponin AIII, timosaponin AI and sarsasapogenin in an intestinal bacterial matrix.

**Figure 6:**
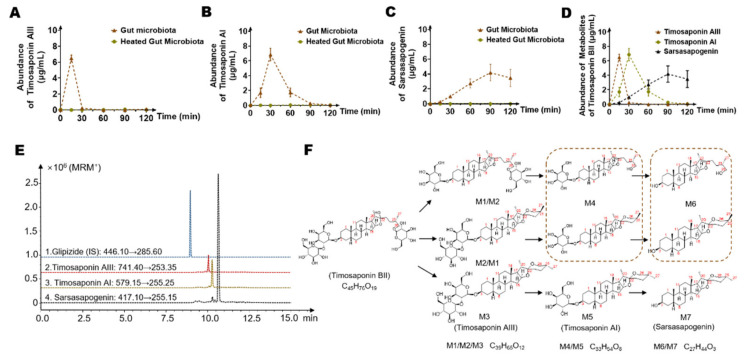
The level of possible timosaponin BII metabolites changed during incubation with rat intestinal bacteria after 0 min, 15 min, 30 min, 60 min, 90 min, and 120 min. (**A**) Metabolic curve of timosaponin AIII. (**B**) Metabolic curve of timosaponin AI. (**C**) Metabolic curve of sarsasapogenin. (**D**) The temporal characteristics of potential timosaponin BII metabolites by gut microbiota. (**E**) Extracted ion chromatogram (EIC) spectra of timosaponin AIII, timosaponin AI, sarsasapogenin and the internal standard (Glipizide). (**F**) Metabolic pathway of timosaponin BII and possible structures of metabolites.

A representative chromatogram for the established method is shown in Figure 6E. The retention times of timosaponin AIII, timosaponin AI and sarsasapogenin were 10.2 min, 10.4 min, and 10.6 min, respectively. The methodology validation was shown in Figures S1 and S2 and in Tables S1–S5. Timosaponin BII was incubated with rat colon contents, and the reaction was terminated by adding three-fold methanol containing an IS at 0 h, 0.25 h, 0.5 h, 1 h, 1.5 h and 2 h. Then, the concentration of three potential metabolites in the system was determined. As shown in Figure 6A–C, timosaponin AIII, representing the monoglucose hydrolyzed metabolites, was first rapidly generated in the system, reaching the highest level (6.49 ± 0.41 μg/mL) at 15 min. Then, timosaponin AIII disappeared rapidly, indicating that timosaponin AIII was not the final product of the intestinal bacteria in this experiment. Timosaponin AI, a metabolite produced by removing two glucosyl groups from the parent drug, also underwent a process of first generation and then disappearance. Timosaponin AI gradually increased from 0–30 min and was then further metabolized from 30–120 min (Figure 6B). Sarsasapogenin was a representative metabolite obtained by cleaving all sugar groups from timosaponin BII. As the incubation time was extended, the content of sarsasapogenin gradually increased, reached a suspected plateau at 90 min and then seemed to decrease slightly, which suggested that sarsasapogenin may experience further unknown metabolism and further research needs to be conducted (Figure 6C). Above all, we concluded the temporal relationships of timosaponin AIII, timosaponin AI and sarsasapogenin in Figure 6D.

In the inactivated control group, none of the three metabolites were detected, indicating that the three substances were produced through metabolism by intestinal bacteria. Overall, we have summarized the possible pathways of timosaponin BII metabolism by gut microbiota, as shown in Figure 6F.

## 3. Discussion

Anemarrhenae Rhizoma is a traditional Chinese medicine commonly used in Chinese medicinal formulae and has the functions of clearing heat, purging fire, nourishing yin and moisturizing dryness. It is traditionally used to treat high fever, polydipsia, internal heat, consumptive thirst, bone steaming and tidal fever. Timosaponin BII is one of the saponins in Anemarrhenae Rhizoma. According to modern pharmacological research, timosaponin BII has anti-Alzheimer’s disease effects and regulates blood glucose [4,6]. Recently, timosaponin BII was in a clinical trial for the treatment of dementia. As an oral natural product, timosaponin BII has low oral bioavailability. However, the effective components of timosaponin BII may not be limited to the prototype drug itself, but the metabolites could play a very important role in its effects. In addition to the impact of the digestive enzymes of the hosts, timosaponin BII administered to the gastrointestinal tract may also interact with the intestinal flora.

In this study, we focused on the characteristics of timosaponin BII metabolism by the gut microbiota. A total of seven possible metabolites of timosaponin BII were identified and may be the hydrolyzed products of the parent drug. The structures and mass spectral fragment pathways of the possible metabolites were analyzed by quadrupole time-of-flight tandem mass spectrometry. Among them, three structures identified are reported in gut microbiota for the first time. At the same time, we sorted out the temporal relationship among the three metabolites, which is important for the study of drug metabolism by intestinal bacteria.

Several possible metabolites may possess pharmacological activities that are not similar to those of the prototype drug. For example, timosaponin AIII is a hydrolyzed metabolite produced by the removal of the β-D-O glucosidic bond at C_26_ in timosaponin BII. Studies have shown that timosaponin AIII can induce the apoptosis of a variety of tumor cells, such as breast cancer and colorectal cancer cells, by activating the ATM/Chk2 and p38 MAPK signaling pathways, inducing autophagy, activating Caspase-4/9, and blocking the cell cycle [2,29,30]. At the same time, timosaponin AIII has also been reported to improve memory function and protect cardiovascular functions [3,7]. It is worth noting that the inhibitory effect on platelet aggregation of timosaponin AIII is stronger than that of timosaponins BII [31]. In addition, timosaponin AI is the metabolite of timosaponin BII produced by glucose removal at C_26_ and C_3_. Studies have shown that timosaponin AI inhibits 5-lipoxygenase activity in vitro, with an IC_50_ of 0.63 μM [32]. Furthermore, timosaponin AI also showed dose-dependent inhibitory activity against DPP-4 [33]. Sarsasapogenin is the ligand remaining after all the glycosides of timosaponin BII have been removed and has strong physiological activities. Sarsasapogenin can instantly increase the oxidative stress in the mitochondria of tumor cells and induce the apoptosis of tumor cells [1]. At the same time, sarsasapogenin can significantly increase the levels of norepinephrine and serotonin in the hypothalamus and hippocampus and can also inhibit the activity of monoamine oxidase, which indicates that it has the potential to treat depression [34]. Thus, the metabolites produced by intestinal flora are likely the sources of diverse pharmacological activities of timosaponin BII.

As we have observed, M1–M7 were de-glycosylated metabolites by the gut microbiota. Some literature has pointed out that gut flora can use glycosides as an energy source [35]. Enzymes with β-glucosidase activity are widely present in intestinal bacteria. For example, the dominant species of intestinal bacteria, such as Bifidobacteria and Lactobacillus, have related enzyme systems [36]. Therefore, the hydrolysis of β-glucosidic bonds by intestinal flora tends to take precedence over the hydrolysis of other glycosidic bonds. Our results showed that timosaponin AIII, a representative metabolite with one less glucose molecule, was preferentially produced by intestinal bacteria, which was consistent with this theory. For breaking non-monoglycosidic bonds, specific enzymes have evolved by certain genera of intestinal bacteria. For example, Lactobacillus acidophilus possesses α-L-rhamnosidase, which enables the hydrolysis of rhamnoside bonds and can be used to deglycosylate rutin and isohesperidin [37,38]. After incubation, timosaponin BII was metabolized to timosaponin AI, sarsasapogenin and their isomers by gut microbiota. Our results indicated that the hydrolysis of oligosaccharide chains by intestinal bacteria may not be done in one step. The glucose moiety on the lactose chain of timosaponin BII is hydrolyzed preferentially, and then the galactose linked to the steroid nucleus is consumed. At the same time, according to the mass spectra of M4–M5, no metabolites produced by preferential removal of the entire lactose chain were detected in our incubation system; that is, no production of 26-O-sarsasapogenin monoglucoside was found, indicating that even though galactose exists simultaneously with glucose on the core skeleton in the form of a monoglycoside, glucose is more easily removed. This may be related to the phenomenon of “glucose inhibition” in microorganisms; that is, glucose is a preferentially used carbon source, and galactose cannot be used as a carbon source at the same time in the presence of high concentrations of glucose because the expression of the genes involved in the metabolism of galactose is inhibited by glucose [39].

In this experiment, there was no obvious interaction between timosaponin BII and liver microsomes or liver homogenates. It has been reported that after oral administration of timosaponin, some glucuronic acid metabolites were found in the urine of rats [40], which may be a result of the Phase II metabolic enzymes in liver microsomes or liver homogenate needing to react with sarsasapogenin, and it is more difficult to directly interact with timosaponin BII. At the same time, there may be few enzymes that can hydrolyze timosaponin BII in liver microsomes or liver homogenate. Differences among species may also explain this result. This also indirectly illustrates the importance of studying the metabolism of timosaponin BII by intestinal flora.

Of course, this study also has certain limitations. For example, the gut microbiota incubation system used in this experiment was derived from the feces of SD rats, and the composition and distribution of the intestinal bacteria among different species or among different individuals of the same species are different, which may lead to dissimilar results. Therefore, validation with different species is needed to further expand the implications of this experiment. At the same time, the digestive enzymes contained in the gastrointestinal tract may also have a metabolic effect on drugs. Differentiating the contributions of intestinal bacteria and other factors to drug metabolism needs to be confirmed by both in vivo experiments and in vitro experiments. Some studies have shown that gut microbiota may metabolize the core skeleton of natural products. For example, flavonoids can be converted into small molecular metabolites of polyphenols by intestinal bacteria [41]. Whether the core of saponins can be metabolized by gut microbiota requires more sensitive analysis methods and more in-depth metabolic research.

In summary, this study focused on the characteristics of timosaponin BII biotransformation by intestinal flora, and HPLC-MS/MS and HPLC/MS-Q-TOF were involved in the identification and quantitation of the metabolites. A total of seven metabolites was identified in the gut microbiota incubation system. The molecular formulas were C_39_H_64_O_13_ ([M + H]^+^, *m*/*z* = 741.4417, M1–M3), C_33_H_54_O_8_ ([M + H]^+^, *m*/*z* = 579.3891, M4, M5) and C_27_H_44_O_3_ ([M + H]^+^, *m*/*z* = 417.3367, M6, M7), respectively. The temporal relationship among metabolites was discussed, which may aid studies on the effective components of timosaponin BII and guide future clinical applications.

## 4. Materials and Methods

### 4.1. Instruments and Reagents

Timosaponin BII (CAS, 136656-07-0; Cat Number, IT0640), timosaponin AIII (CAS, 41059-79-4; Cat Number, IT0630), Sarsasapogenin (CAS, 126-19-2; Cat Number, SS8160), and Glipizide (CAS, 29094-61-9; Cat Number, SG8680) were purchased from Solarbio Life Sciences Co., Ltd. (Beijing, China). Timosaponin AI (CAS, 68422-00-4; Cat Number, B21655) was purchased from Shanghaiyuanye Bio-Technology Co., Ltd. The purity of all standard reagents was qualified for quantitative analysis. HPLC grade methanol and LC/MS grade formic acid were purchased from Fisher Scientific (Fair Lawn, NJ, USA), and GR grade ammonia was purchased from Shanghai Macklin Biochemical Co., Ltd. Male SD rat liver microsome was purchased from Research Institute for Liver Diseases Co., Ltd (Shanghai, China). The High-Performance Liquid Chromatograph (HPLC) was purchased from Shimadzu Corporation (Kyoto, Japan), and coupled with a triple quadrupole mass spectrometer from Shimadzu Corporation (Kyoto, Japan), LCMS-8060, used for the quantitative detection of timosaponin BII and its metabolites. Another HPLC tandem quadrupole time-of-flight mass spectrometer from Shimadzu Corporation (Kyoto, Japan), LCMS-9030, was applied for the qualitative identification and structural analysis of metabolites of timosaponin BII. A shaking incubator was purchased from Longyue Instrument Co., Ltd. (Shanghai, China). A WH-681 vortex mixer was purchased from Jintan Shenglan Instrument Manufacturing Co., Ltd (Jintan, China). The refrigerated high-speed centrifuge was purchased from Eppendorf (Hamburg, Germany).

### 4.2. Animals

Seven Sprague Dawley adult male rats (200–300 g) were purchased from SPF Biotechnology Co., Ltd. (Beijing, China). All animals had free access of food and water, and were housed in a ventilated room with a circulation of 12 h of light and 12 h of darkness. The temperature is maintained at 20–24 °C and humidity 40–60%. The rats were fasted for 12 h and allowed to drink freely before the experiment. This study was approved by the Experimental Animal Ethics Committee of the Chinese Academy of Medical Sciences and Peking Union Medical College (No. 00003402, date of approval, 24 June 2020), and strictly followed the instruction of Organizational Guidelines and Ethics Guidelines of the Experimental Animal Ethics Committee.

### 4.3. Determination of Timosaponin BII by LC-MS/MS

The quantitative detection of timosaponin BII and possible metabolites was performed using LCMS-8060 equipped with an ESI ion source. An XBridge^®^ BEH C8 column (75 × 3.0 mm, 2.5 μm, Waters, Wexford, Ireland) was applied for separation of the analytes. The flow rate was 0.4 mL/min, and the temperature of column oven was 40 °C. The injection volume was 10 μL. The mobile phase used was aqueous ammonia: water (0.075:100, *v*/*v*) as mobile phase A and methanol as mobile phase B. Gradient elution condition (B%) was as follows: 0.01 min, 30%→3.00 min, 30%→6.00 min, 50%→8.00 min, 95%→10.00 min, 95%→12.00 min, 30%→17.00 min, stop. The MRM mode was used for detection by the mass spectrometer, with mass transitions for timosaponin BII (negative MRM) of 919.40→757.40 (Q1 Pre Bias: 28.0 V, CE: 45.0 V, Q3 Pre Bias: 28.0 V, Dwell Time: 50 msec), timosaponin AIII (positive MRM) of 741.40→253.35 (Q1 Pre Bias: −20.0 V, CE: −32.0 V, Q3 Pre Bias: −11.0 V, Dwell Time: 50 msec), timosaponin AI (positive MRM) of 579.15→255.25 (Q1 Pre Bias: −24.0 V, CE: −27.0 V, Q3 Pre Bias: −17.0 V, Dwell Time: 50 msec), sarsasapogenin (positive MRM) of 417.10→255.15 (Q1 Pre Bias: −20.0 V, CE: −25.0 V, Q3 Pre Bias: −20.0 V, Dwell Time: 50 msec), and the internal standard (IS, glipizide) of 446.10→285.60 (positive MRM, Q1 Pre Bias: −16.0 V, CE: −25.0 V, Q3 Pre Bias: −19.0 V, Dwell Time: 50 msec) and 444.25→319.10 (negative MRM, Q1 Pre Bias: 18.0 V, CE: 18.0 V, Q3 Pre Bias: 24.0 V, Dwell Time: 50 msec), respectively. The mass spectrometer parameters were set as follows: nebulizer gas, 3.0 L/min; heating gas, 10 L/min; interface temperature, 300 °C; DL temperature, 250 °C; heat block temperature, 400 °C; drying gas, 10 L/min; interface voltage, −4.5 kV; and CID gas pressure, 270 kPa.

All the samples were maintained at 4 °C before injection. Sample preparation will be mentioned in Section 4.5 and Section 4.6.

### 4.4. Identification of the Metabolites of Timosaponin BII by LC/MS-Q-TOF

The qualitative identification of timosaponin BII and possible metabolites was performed using LCMS-9030 equipped with an ESI ion source. A Shim-pack GIST C18 column (100 × 2.1 mm, 2.0 μm, Shimadzu, Kyoto, Japan) was applied for separation of the analytes. The flow rate was 0.4 mL/min, and the temperature of column oven was 40 °C. The injection volume was 7 μL. The mobile phase used was formic acid: water (0.05:100, *v*/*v*) as mobile phase A and methanol as mobile phase B. Gradient elution condition (B%) was as follows: 0.01 min, 30%→3.00 min, 30%→6.00 min, 50%→8.00 min, 95%→10.00 min, 95%→12.00 min, 30%→17.00 min, stop. The mass spectrometer parameters were set as follows: nebulizer gas, 3.0 L/min; heating gas, 10 L/min; interface temperature, 300 °C; desolvation temperature, 526 °C; DL temperature, 250 °C; heat block temperature, 400 °C; drying gas, 10 L/min; interface voltage, 4.5 kV (+)/−3.0 kV (−); CID gas pressure, 230 kPa; and detector voltage, 2.34 kV. The MS mode (both positive and negative) was used to scan the possible metabolites with mass range of 100.0000 to 1000.0000. The DDA mode (both positive and negative) was applied with a mass range of 50.0000 to 1000.0000 and collision energy of 35 ± 17 V.

### 4.5. In Vitro Incubation of Timosaponin BII with Gut Microbiota

After 7 SD rats were sacrificed, the colon contents were collected and added to the sterilized anaerobic medium (Solarbio Life Sciences Co., Ltd., Beijing, China) at a ratio of 1.0 g:20 mL, and stirred gently. After filtering, the culture medium containing gut microbiota (mixed medium) was placed in a N_2_ atmosphere, and pre-incubated at 37 °C for 60 min before use. Accurately weigh 1.0 mg of timosaponin BII and dissolve with methanol to obtain a solution of 1 mg/mL. The incubation system consists of 10 μL of timosaponin BII in methanol (1 mg/mL), and 990 μL of mixed medium. The incubation was conducted in a 37 °C, 200 rpm shaking incubator. The incubation system must be maintained completed in an anaerobic environment during the experiment. In the first incubation experiment, the timosaponin BII and mixed medium were incubated for 0, 1, 2, 6, 12, and 24 h, respectively. In the second incubation experiment, the drugs and mixed medium were incubated for 0, 0.25, 0.5, 1, 1.5, and 2 h, respectively. In addition, the negative control group was introduced consisting of twice boiled mixed medium incubated with the same amount of timosaponin BII. The termination reaction was carried out by adding 3-fold volume of 100 ng/mL glipizide methanol solution (IS), shaking it evenly and then precipitating the protein. After each sample was centrifuged at 13,400× *g* rpm in a 4 °C refrigerated centrifuge for 10 min, 10 μL of supernatant was injected for LC-MS/MS analysis, and 7 μL of supernatant was injected for LC/MS-Q-TOF analysis.

Then, 1 mg/mL of timosaponin BII methanol solution was gradually diluted to a series of stock solutions with concentrations of 500 μg/mL, 100 μg/mL, 50 μg/mL, 10 μg/mL, 5 μg/mL, 1 μg/mL, and 0.5 μg/mL, respectively. Timosaponin BII standard samples were composed of a series of 10 μL of timosaponin BII stock solutions and 990 μL of inactivated medium, for quantification of the metabolism of timosaponin BII. A mixed solution containing 1 mg/mL of timosaponin AIII, 1 mg/mL of timosaponin AI, and 1 mg/mL of sarsasapogenin was prepared, respectively. Methanol was gradually added to obtain a series of mixed stock solutions of 500 μg/mL, 100 μg/mL, 50 μg/mL, 10 μg/mL, 5 μg/mL, 1 μg/mL and 0.5 μg/mL. Mixed standard samples were composed of a series of 10 μL mixed stock solutions and 990 μL inactivated medium, for quantification of potential metabolites of timosaponin BII. The rest of the sample processing steps were the same as above.

### 4.6. In Vitro Incubation of Timosaponin BII with Liver Microsomes and Liver Homogenate

The liver microsome incubation system was consisted of the following: 5 μL SD rat liver microsomes (20 mg/mL), 2 μL timosaponin BII (1 mM), 20 μL of NADPH, and 0.05 mM Tris/HCl (pH = 7.4), with a total volume of 200 μL. The incubation was conducted in a shaking incubator at 37 °C and 800 rpm with supply of oxygen. After the incubation, 3-fold volume of glipizide methanol solution (IS, 100 ng/mL) was added to the incubation system and mixed to stop the reaction at 0, 0.25, 0.5, 1, 1, 5, and 2 h. After centrifugation at 13,400× *g* rpm in a refrigerated centrifuge at 4 °C for 10 min, 10 μL of the supernatant was injected for LC-MS/MS analysis.

Liver homogenate was prepared by homogenizing freshly collected SD rat livers and adding ice-cold normal saline with weight/volume = 1:3. The liver homogenate incubation system was consisted as follows: 2 μL timosaponin BII (1 mM), 198 μL of freshly prepared liver homogenate, with a total volume of 200 μL.

Incubation was conducted in a shaking incubator at 37 °C and 800 rpm with supply of oxygen. After the incubation, 3-fold volume of glipizide methanol solution (IS, 100 ng/mL) was added to the incubation system and mixed to stop the reaction at 0, 0.25, 0.5, 1, 1, 5, and 2 h. After centrifugation at 13,400× *g* rpm in a refrigerated centrifuge at 4 °C for 10 min, 10 μL of the supernatant was injected for LC-MS/MS analysis.

Then, 1 mg/mL of timosaponin BII methanol solution was gradually diluted to a series of stock solutions with concentrations of 500 μg/mL, 100 μg/mL, 50 μg/mL, 10 μg/mL, 5 μg/mL, 1 μg/mL, and 0.5 μg/mL, respectively. Timosaponin BII standard samples were composed of a series of 2 μL of timosaponin BII stock solutions and 198 μL of 0.05 mM Tris/HCl (pH = 7.4) or 198 μL of inactivated liver homogenate for the detection of timosaponin BII. The rest of the sample processing steps were the same as above.

### 4.7. Statistical Analysis

Mass spectrum data acquisition and subsequent data processing were performed with Shimadzu LabSolutions (version 5.89, Kyoto, Japan) of quantitative analysis on LCMS-8060 and LabSolutions Insight™ Explore (Kyoto, Japan) of qualitative analysis on LCMS-9030. Two-tailed ANOVA and Student’s *t*-test were used for statistical analysis with GraphPad Prism Version 5 (GraphPad Software, San Diego, CA, USA). Data were expressed as the mean ± standard deviation (SD), and *p* values less than 0.05 were considered statistically significant.

## Figures and Tables

**Table 1:** Characteristics of Timosaponin BII metabolites in gut microbiota by LC/MS-Q-TOF.

Metabolites	Retention Time (min)	Reaction *	Predicted Molecular Weight	Molecular Formula	Fragment Characteristics	
					MS1/[M + H]^+^	MS/MS
**M1**	9.9	−Glc **	740.93	C_39_H_65_O_12_	741	705, 579, 561, 435, 273, 255
**M2**	10.1	−Glc	740.93	C_39_H_65_O_12_	741	705, 579, 561, 435, 273, 255
**M3**	10.2	−Glc	740.93	C_39_H_65_O_12_	741	705, 579, 561, 435, 273, 255
**M4**	10.3	−2Glc	578.38	C_33_H_54_O_8_	579	533, 435, 273, 255
**M5**	10.6	−2Glc	578.38	C_33_H_54_O_8_	579	533, 435, 273, 255
**M6**	10.9	−2Glc-Gal ***	416.65	C_27_H_44_O_3_	417	273, 255
**M7**	11.3	−2Glc-Gal	416.65	C_27_H_44_O_3_	417	273, 255

* Reaction refers to the process to produce metabolites; ** Glc refers to the glucose moiety; *** Gal refers to the galactose moiety.

## Data Availability

The data in this study are available in this article.

## Supplementary Materials

The following are available online. Figure S1: Specificity of established method. (A) the mass spectra of the blank incubation culture of gut microbiota. (B) the mass spectra of the culture spiked with timosaponin BII, timosaponin AIII, timosaponin AI, sarsasapogenin and IS; Figure S2: Representative standard curve of timosaponin BII (A), timosaponin AIII (B), timosaponin AI (C) and sarsasapogenin (D). Table S1: Method sensitivity and linear range of timosaponins; Table S2: Accuracy and recision of the method for determination of timosaponins (n = 5); Table S3: Recovery of the method for determination of timosaponins (n = 5); Table S4: Stability at room temperature for 4 h before treatment (n = 5); Table S5: Stability in the autosampler for 24 h at 4 °C post-treatment (n = 5).

## Sample Availability

Samples of the compounds timosaponin BII, timosaponin AIII, timosaponin AI and sarsasapogenin are available from the authors.
