# Supplementary material for: Biotransformation of Timosaponin BII into Seven Characteristic Metabolites by the Gut Microbiota
Source: Molecules. 2021 Jun 24;26(13):3861. doi: 10.3390/molecules26133861 (PMC8270264; doi:10.3390/molecules26133861)
Supplement: Supplementary file 1 [file molecules-26-03861-s001.zip › molecules-1247071-supplementary.pdf]

Biotransformation of Timosaponin BII into Seven Characteristic Metabolites by the Gut Microbiota

Guo-Ming Dong [1,†], Hang Yu [2,†], Li-Bin Pan [2], Shu-Rong Ma [2], Hui Xu [2], Zheng-Wei Zhang [2], Pei Han [2], Jie Fu [2],
Xin-Yu Yang [2], Adili Keranmu [2], Hai-Tao Niu [3], Jian-Dong Jiang [2,*] and Yan Wang [2,*]

[1] Beijing Hwellso Pharmaceutical Co., Ltd., Beijing 100044, China; qcdgm@126.com
[2] State Key Laboratory of Bioactive Substance and Function of Natural Medicines, Institute of Materia Medica, Chinese Academy of Medical Sciences/Peking Union Medical College, Beijing 100050, China; yuhang@imm.ac.cn (H.Y.); panlibin@imm.ac.cn (L.-B.P.); mashurong@imm.ac.cn (S.-R.M.); xuhui@imm.ac.cn (H.X.); zhangzhengwei@imm.ac.cn (Z.-W.Z.); hanpei@imm.ac.cn (P.H.); fujie@imm.ac.cn (J.F.); xinyuy20@mails.jlu.edu.cn (X.-Y.Y.); adili117@163.com (A.K.);
[3] School of Medicine and Institute of Laboratory Animal Sciences, Jinan University, Guangzhou Key Laboratory of Germ-free Animals and Microbiota Application, Guangzhou 510632, China; htniu@jnu.edu.cn
* Correspondence: jiang.jdong@163.com (J.-D.J.); wangyan@imm.ac.cn (Y.W.); Tel.: +86-10-831-600-05 (J.-D.J.); +86-10-6316-5238 (Y.W.); Fax: +86-10-630-177-57 (J.-D.J.); +86-10-6316-5238 (Y.W.)
† These authors contributed equally to this work.

Supplementary Materials

Validation of timosaponins in gut microbiota by LC-MS/MS

Calibration standards and QC preparation

Prepare a mixed solution containing 1 mg/mL of 1 mg/mL of timosaponin BII, timosaponin AIII, 1 mg/mL of timosaponin AI, and 1 mg/mL of sarsasapogenin. And gradually add methanol to obtain a series of mixed stock solutions of 500 μg/mL, 100 μg/mL, 50 μg/mL, 10 μg/ mL, 5 μg/mL, 1 μg/mL and 0.5 μg/mL. Mixed standard samples were composed of a series of 10 μL mixed stock solutions and 990 μL inactivated medium. The standard curve concentrations were as follows: 5, 10, 50, 100, 500, 1000, 5000 and 10000 ng/mL. The concentrations of QCs were 10 ng/mL (for low concentration of quality control, LQC), 500 ng/mL (median concentration of quality control, MQC), and 8000 ng/mL (high concentration of quality control, HQC). The sample processing steps were carried out by adding 3-fold volume of 100 ng/mL glipizide methanol solution (IS), shaking it evenly and then precipitating the protein. After each sample was centrifuged at 13,400 rpm in a 4℃ refrigerated centrifuge for 10 min, 10 μL of supernatant was injected for LC-MS/MS analysis.

Method validation

Method validation was carried out by determination of specificity, linearity, intra- and inter-day accuracy and precisions, recovery and stability according to the validation guidelines of biological sample analysis by Chinese State Food and Drug Administration.

Specificity

The specificity of the method was determined by analyzing the blank incubation culture of gut microbiota and the culture spiked with timosaponin BII, timosaponin AIII, timosaponin AI, sarsasapogenin and IS, respectively.

Linearity and Sensitivity

Linearity was determined by plotting the peak area ratio of analytes to IS against the theoretical concentrations of the standards with weighted (*1/c*) least square linear regression. Lower limit of determination (LLOD) was defined as the concentration at which the signal-noise ratio was 3:1. Lower limit of quantification (LLOQ) was defined as the lowest concentration of the standard curve.

Precision and Accuracy

The inter- and intra-day precision and accuracy were carried out by analyzing repeated quality control samples (LQCs, MQCs, HQCs, n=5) on three consecutive days. The precision and accuracy were expressed as the relative standard deviation (RSD, %) and Accuracy (%), respectively.

Recovery

Recovery was calculated by comparing the peak area of five replicates of QC samples (LQCs, MQCs, and HQCs) with the peak area of the post-treatment spiked samples.

Stability

The stability of the analytes in gut microbiota was investigated by analyzing the pretreated QC samples stored at room temperature for 4h and the post-treated QC samples placed in the autosampler at 4℃ for 24 h.

Results

The mass spectra of timosaponin BII, timosaponin AIII, timosaponin AI, sarsasapogenin and the internal standard (glipizide) in incubated culture were shown in Figure S1B and the retention times of timosaponin BII, timosaponin AIII, timosaponin AI, sarsasapogenin and the internal standard were 9.5 min, 10.2 min, 10.4 min, 10.6 min and 9.1 min, respectively. And significant interference was not found in the blank incubation culture (Figure S1A), which indicates the excellent specificity of the method.

The calibration curves of the analytes were linear, with correlation coefficients r^2 >0.99, and the linear range of timosaponin BII, timosaponin AIII, timosaponin AI and sarsasapogenin was 5-10000 ng/mL (Table S1, Figure S2). The LLOD of timosaponin BII and 3 metabolites was 1 ng/mL, and the LLOQ was 5 ng/mL (Table S1).

Accuracy and precision results showed that the measured values were within the ±15% deviation and met the requirements (Table S2)

The recovery timosaponin BII, timosaponin AIII, timosaponin AI and sarsasapogenin was in the range of 99.06-102.7%, 97.61-100.2%, 96.96-100.4%, and 98.33-101.1%, respectively, suggesting that the sample processing method was proper for the extraction of timosaponin BII, timosaponin AIII, timosaponin AI and sarsasapogenin (Table S3).

Results of stability showed that timosaponin BII, timosaponin AIII, timosaponin AI and sarsasapogenin were stable under the analytical process of samples (Table S4, S5).

These data demonstrated that the developed method was reliable and reproducible for the quantitative analysis of timosaponin BII with its metabolites (timosaponin AIII, timosaponin AI and sarsasapogenin) in gut micobiota.

Table S1. Method sensitivity and linear range of timosaponins

	Timosaponin BII	Timosaponin AIII	Timosaponin AI	Sarsasapogenin
**Instrumental LLOD (ng/mL)**	1	1	1	1
**Instrumental LLOQ (ng/mL)**	5	5	5	5
**Linear range (ng/mL)**	5-10000	5-10000	5-10000	5-10000

Table S2. Accuracy and recision of the method for determination of timosaponins (n=5)

		Timosaponin BII			Timosaponin AIII			Timosaponin AI			Sarsasapogenin		
**Batch**		Mean (ng/mL)	Accuracy (%)	RSD (%)	Mean (ng/mL)	Accuracy (%)	RSD (%)	Mean (ng/mL)	Accuracy (%)	RSD (%)	Mean (ng/mL)	Accuracy (%)	RSD (%)
**1**	LQC	10.17	101.7	4.527	10.06	100.6	1.277	10.03	100.3	0.7051	10.29	102.9	3.491
	MQC	500.4	100.1	1.982	503.6	100.7	1.593	498.3	99.65	1.580	496.8	99.36	1.871
	HQC	8053	100.7	2.871	8024	100.3	0.8071	7998	99.97	0.9589	8063	100.8	1.771
**2**	LQC	9.95	99.49	4.330	10.20	102.0	4.825	10.32	103.2	3.635	10.13	101.3	1.408
	MQC	489.7	97.94	3.743	516.3	103.3	3.820	497.8	99.56	1.537	499.1	99.81	3.651
	HQC	8041	100.5	3.212	8097	101.2	2.482	7989	99.86	2.864	7958	99.48	2.512
**3**	LQC	9.90	98.97	1.864	10.07	100.7	5.138	9.74	97.42	2.965	10.18	101.8	3.073
	MQC	497.0	99.40	3.957	504.1	100.8	2.637	498.7	99.74	1.884	505.6	101.1	2.821
	HQC	7981	99.76	4.742	8030	100.4	3.935	8117	101.5	1.800	8167	102.1	1.668
**int**	L	10.01	100.1	3.	10.11	101.1	3.88	10.03	100.3	3.53	10.20	102.0	2.688

**er-**	QC			717			9			8			
**day**	MQC	495.7	99.14	3.227	508.0	101.6	2.900	498.3	99.65	1.552	500.5	100.1	2.774
	HQC	8025	100.3	3.442	8050	100.6	2.559	8035	100.4	2.021	8063	100.8	2.159

Table S3. Recovery of the method for determination of timosaponins (n=5)

		LQC	MQC	HQC
Timosaponin BII	Recovery (%)	102.7	101.1	99.06
	RSD (%)	4.507	4.341	4.508
Timosaponin AIII	Recovery (%)	97.61	101.1	100.2
	RSD (%)	8.808	2.741	4.485
Timosaponin AI	Recovery (%)	100.4	96.96	98.60
	RSD (%)	5.234	5.283	4.166
Sarsasapogenin	Recovery (%)	98.33	99.87	101.1
	RSD (%)	7.627	6.553	5.528

Table S4. Stability at room temperature for 4 h before treatment (n=5)

		LQC	MQC	HQC
Timosaponin BII	Accuracy (%)	102.4	103.0	100.2
	RSD (%)	2.395	1.764	4.951
Timosaponin AIII	Accuracy (%)	105.3	97.12	100.7
	RSD (%)	8.276	2.314	5.725
Timosaponin AI	Accuracy (%)	97.91	99.31	101.9
	RSD (%)	5.120	7.111	4.652
Sarsasapogenin	Accuracy (%)	99.45	101.6	101.8
	RSD (%)	4.215	4.379	2.515

Table S5. Stability in the autosampler for 24h at 4℃ post-treatment (n=5)

		LQC	MQC	HQC
Timosaponin BII	Accuracy (%)	103.7	99.24	98.78
	RSD (%)	5.563	2.790	5.127
Timosaponin AIII	Accuracy (%)	98.94	103.0	98.56
	RSD (%)	9.835	4.321	3.133
Timosaponin AI	Accuracy (%)	102.4	97.61	100.4
	RSD (%)	6.692	3.236	2.625
Sarsasapogenin	Accuracy (%)	99.51	102.6	98.24
	RSD (%)	7.857	5.780	1.890

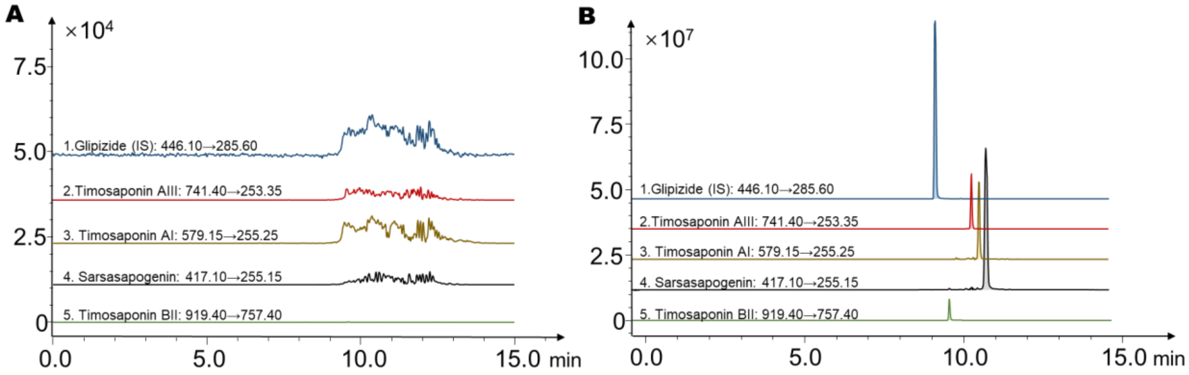

Figure S1. Specificity of established method. (A) the mass spectra of the blank incubation culture of gut microbiota. (B) the mass spectra of the culture spiked with timosaponin BII, timosaponin AIII, timosaponin AI, sarsasapogenin and IS.

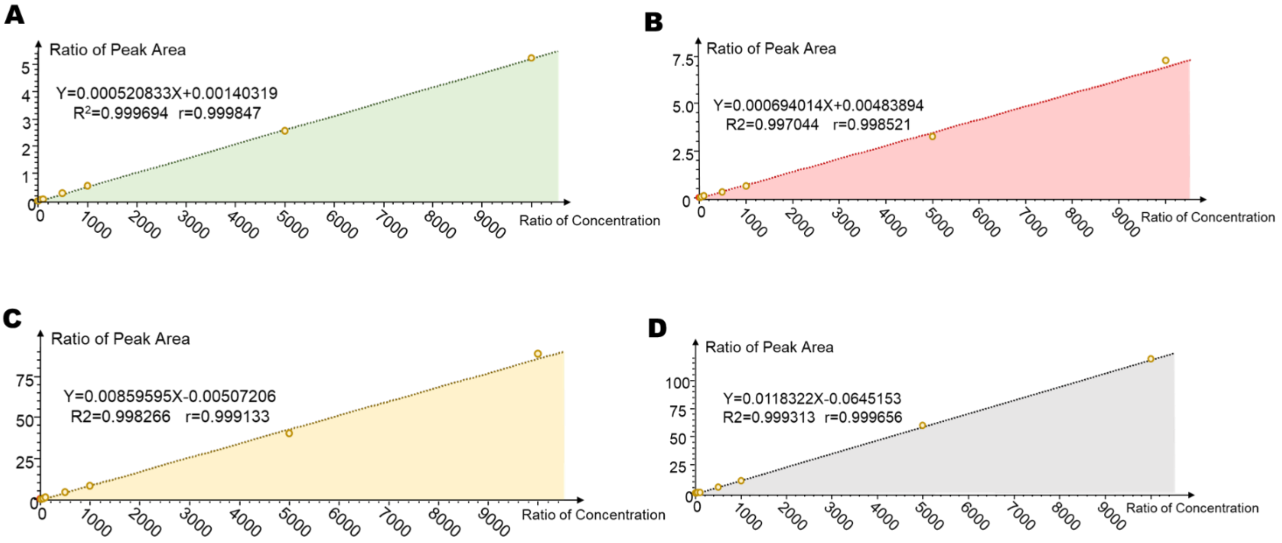

Figure S2. Representative standard curve of timosaponin BII (A), timosaponin AIII (B), timosaponin AI (C) and sarsasapogenin (D).
